# Partial Spinous Process Decompression in Baastrup’s Disease: A Case Report and Literature Review

**DOI:** 10.7759/cureus.34070

**Published:** 2023-01-22

**Authors:** Felix Corr, Dustin Grimm, Ralf D Rothoerl

**Affiliations:** 1 Department of Neurosurgery and Spine Surgery, Isarklinikum, Munich, DEU

**Keywords:** baastrup’s disease, kissing spine disease, computed tomography, low back pain, spinal diseases

## Abstract

Baastrup’s disease represents a frequent, primarily radiological phenomenon on imaging studies of the spine. Nevertheless, it can present as a rare, symptomatically relevant pathology that implies a therapeutic consequence. Yet, there is little evidence and agreement on a consistent treatment strategy in the current literature. Here, we present the case of a 46-year-old man who presented with chronic, persistent midline back pain that was relieved by flexion and aggravated by spinal extension. Extensive imaging studies, including computed tomography, magnetic resonance imaging, and single-photon emission computed tomography confirmed the close approximation of the spinous processes at the levels L4-L5 and L5-S1. Clinically symptomatic isolated Baastrup’s disease was confirmed by a local anesthetic infiltration test. As conservative treatment options failed, partial resection of the spinous processes was performed. Conservative treatment, including analgesics and physical therapy, represents the initial treatment approach for Baastrup’s disease. When clinical features of Baastrup’s disease are present, differential diagnoses have been excluded, and conventional therapy has been exhausted surgical decompression with low surgical risk and good prognosis may be indicated after careful consideration of the indications.

## Introduction

Low back pain is a common condition in the aging society and represents one of the leading causes of disability worldwide [1]. Less frequently, the spinous processes as posterior elements of the spinal vertebrae are considered to be the pathological origin of back pain. Baastrup’s disease, also known as “kissing spines,” refers to a frequently observed pathology of the vertebral column, which was first described as a radiological sign in 1933 as a cause for postural back pain [2]. Although the exact etiology is not yet properly understood, Baastrup’s disease is characterized by close approximation and contact of the spinous processes, leading to reactive sclerosis, further degeneration, and irritation of the paraspinal soft tissues [2].

Baastrup’s disease is a frequently observed radiological phenomenon [3]. However, it represents an essential but often underdiagnosed and missed differential diagnosis of lumbar back pain [4,5]. During the physical examination, localized pain on palpation of the spinous processes, with additional aggravation on extension and relief on flexion, comprise pathognomic clinical features [5]. Diagnostic workup includes dynamic flexion-extension radiographs of the lumbar spine [6]. Computed tomography (CT) and magnetic resonance imaging (MRI) are necessary imaging modalities for the workup of differential diagnoses [5]. Targeted interspinous infiltration using local anesthetics combined with cortisone may achieve local symptom relief [7]. Adequate pain management along with physiotherapy are the basics of conservative treatment options [6].

So far, there is no uniformly defined treatment strategy for Baastrup’s disease that can be used as a therapeutic escalation when conservative treatment methods fail [7]. Despite being an often missed pathology with little available scientific evidence regarding invasive treatment modalities, there is a need for a potentially curative treatment approach [8]. Hence, considering the lack of evidence for surgical approaches, this article emphasizes the role of partial surgical resection.

## Case presentation

A 46-year-old man presented with chronic, ongoing lumbar back pain for two years. The pain was localized to the midline with no paravertebral pain. On physical examination, palpation of the spinous processes L4, L5, and S1 precipitated pain. He reported the pain intensity to be a six out of ten on the numeric rating scale (NRS). The patient noted worsening pain with lordosis and relief with kyphosis. There was no evidence of radiculopathy. Muscle strength and sensation were normal in the lower extremities. No hypo- or hyperreflexia was noted. Sacroiliac joint provocative tests were unremarkable.

Previously conservative management included analgesics and physical therapy. In the past, dual thermal ablation of the right sacroiliac joint and, most recently, single denervation of the left sacroiliac joint for chronic sacroiliac joint arthralgias were performed. Platelet-rich plasma was infiltrated bilaterally into the sacroiliac joints. In addition, the facet joints were ablated twice at levels L3-S1 using percutaneous radiofrequency therapy and infiltrated with local anesthetics and cortisone. There were no other pre-existing medical conditions besides a sequestrectomy at the L5/S1 level for medial disc herniation in the past. Social and family history were unremarkable.

**Figure 1:**
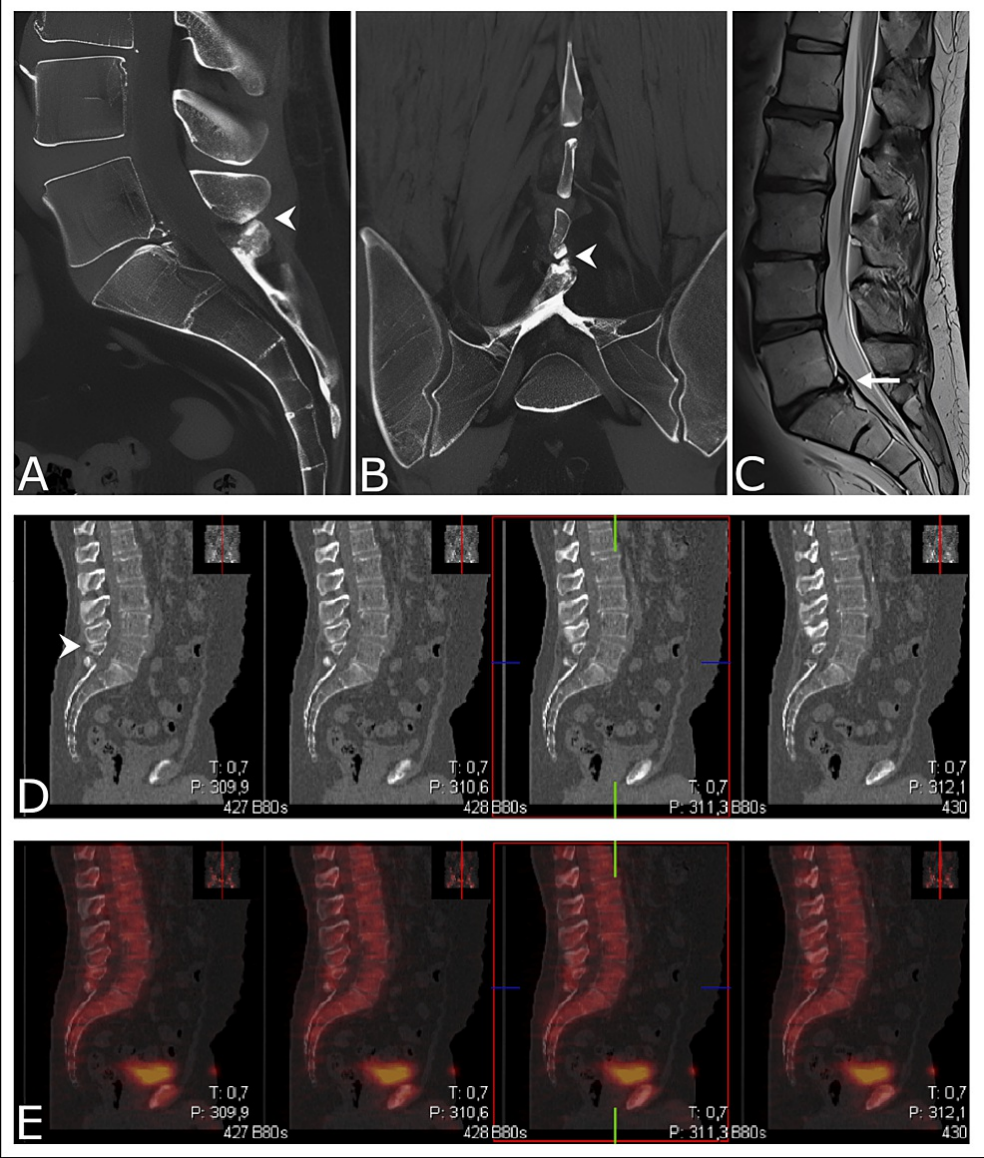
Imaging modalities for diagnostic workup in Baastrup’s disease. (A) Sagittal CT of the lumbosacral junction. The arrow indicates the close relationship of the spinous process L5 and S1 with reactive sclerosis of the opposing interspinous surfaces. A reduced interspinous space of spinous processes L4-L5 is observed. (B) Corresponding coronal CT. The arrow indicates the approximation and contact of the adjacent spinous processes of L5 and S1. (C) T2-weighted sagittal MRI of the lumbosacral junction. Minor degenerative changes and postoperative changes at the L5-S1 segment (long arrow) are evident. (D) The contact of the L4 and L5 spinous processes is visible (arrow). (E) SPECT data do not demonstrate any relevant tracer uptake. CT = computed tomography; MRI = magnetic resonance imaging; SPECT = single-photon emission computed tomography

Diagnostic workup included MRI and CT scan. Single-photon emission computed tomography (SPECT) data were available from previous examinations (see Figure 1). The CT particularly demonstrated the close approximation of spinous processes L5 and S1. Reactive cortical sclerosis at L4-L5 and L5-S1 was noted. Similarly, MRI demonstrated the approximation of the interspinous spaces between L4 and S1 without any signs of accompanying pathological changes. On SPECT, no increased uptake could be observed.

The diagnosis was confirmed by fluoroscopic-guided interspinous infiltration at the levels L4-L5 and L5-S1 with 0.5% ropivacaine and methylprednisolone. Following infiltration, the pain improved by more than 90% for six hours, thereby confirming the pathological site of origin.

**Figure 2:**
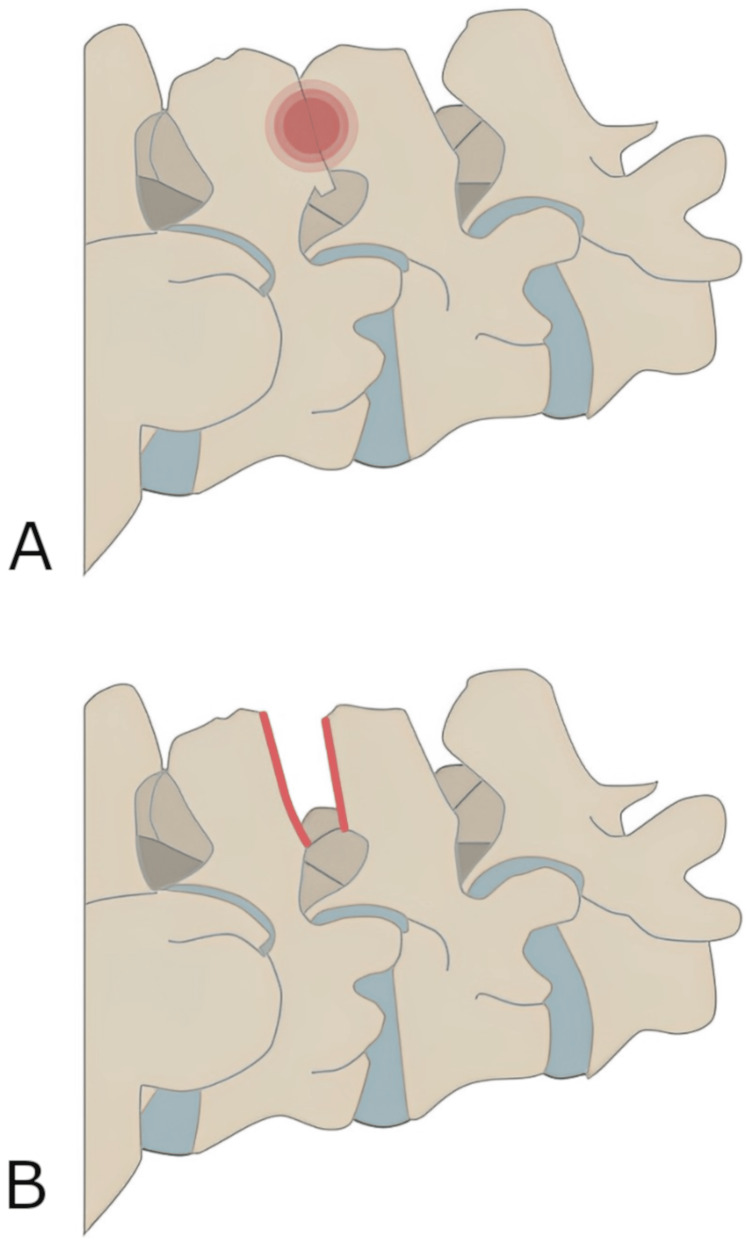
Graphic illustration of the partial surgical resection of the one-third superior and inferior portions of the spinous processes L4-L5. (A) Close approximation and contact of spinous processes leading to irritation (red circle). (B) Segmental spinous processes have adequate distance following partial resection. The identical procedure was performed for the adjacent segment L5-S1. Permission has been granted from Miles Rothoerl.

Following the initial diagnostic interspinous injection, the patient received two additional injections to effectively manage the pain associated with Baastrup’s disease. Although the infiltrations decreased the pain, they only lasted for a few hours. Because conservative methods and infiltrative therapy had been exhausted in this patient, the indication for surgical decompression was given. The procedure was performed under general anesthesia. The patient was placed in a prone position on a fluoroscopic radiolucent operating table. The spinous processes of the lumbar vertebral bodies L4-S1 were visualized using a dorsal midline approach. Surgical decompression of both spinous processes was performed by wedge excision of the one-third superior and inferior portions using a piezoelectric cutting instrument, with additional high-speed drilling of the spinous process margins (see Figure 2). Bone wax was used as the hemostatic agent for bony bleeding. The wound was closed in a layered fashion.

The patient was discharged home two days following surgery. Immediately postoperatively, pain intensity was reported as two out of ten points on the NRS. At discharge, the patient was pain-free, which persisted until the recent six-month follow. At present, the patient continues to be on follow-up.

## Discussion

The close approximation of adjacent spinous processes characterizes Baastrup’s disease. Radiological evidence of pathoanatomical changes does not necessarily implicate a therapeutic sequence. However, in some patients, treatment can be indicated for clinically isolated symptoms associated with morphologic changes in imaging studies and a positive confirmatory infiltration test, as in this case. When conservative treatment fails, we demonstrated that partial resection of the affected spinous processes contributes to pain relief, thus providing an effective surgical alternative with low complications.

Especially in an aging society, degenerative radiological changes of the spine are a common observation [9]. Concerning Baastup’s disease, pathological imaging features were found in approximately 81% of patients over 80 years of age [3]. The most frequently affected segment of the lumbar spine represents the L4-L5 level, followed by L3-L4 and L5-S1 [3,10]. Mainly one to two segments are affected (35.6% vs. 30.5%, respectively) [3]. In our patient, two segments were affected, which is less commonly observed, but did not seem to influence the surgical outcome.

Diagnostic workup includes various imaging modalities. The approximation of the spinous processes along with sclerosis of the articulating surfaces can already be detected by dynamic flexion-extension radiographs of the lumbar spine and thus represents one of the first-line imaging modalities due to its overall low cost, wide availability, and low ionizing radiation dose [5]. CT represents the gold standard for diagnosis by providing higher resolution and, thus, detailed visualization of bony anatomy [5]. Additionally, MRI can be acquired to exclude differential pathologies or accompanying degenerative conditions [5]. CT and MRI were performed in our patient to obtain a high-resolution reconstruction of the bony anatomy in different planes and to detect musculo-ligamentous or other concomitant changes by MRI. SPECT data were available from previous examinations at another institution, which we consulted for co-evaluation.

Overall, although many modalities can be exhausted, it raises the possibility of overdiagnosing or overimaging [11]. As in our case, interventional and diagnostic imaging modalities such as SPECT imaging were extensively exhausted in the patient in other departments, highlighting that Baastrup’s disease is often underdiagnosed or misinterpreted [8]. Regarding other imaging modalities, studies demonstrate the value of SPECT or positron emission tomography-computed tomography imaging in the setting of Baastrup’s disease to distinguish between other pathologies (e.g., spinal bone metastasis) or identify the site of inflammatory pathology [12,13]. It may, therefore, provide differential diagnostic information in unclear circumstances, but it does not necessarily have to be considered in the first-line diagnostic approach.

Regarding conservative treatment, adequate analgesia along with physical therapy should be started as the first-line treatment strategy [5]. Infiltration of the affected spinous with a local anesthetic and cortisone can be helpful as a confirmatory diagnostic test, on the one hand, and as an intermittent treatment modality, on the other [7]. However, the latter usually provides only short-term symptom relief, as in cases of severe approximation of the spinous processes, the underlying etiology is not eliminated but only treated symptomatically [5].

In addition to the bi-segmental Baastrup’s disease in the caudal segments and extended imaging, our patient presented clinically with a purely isolated Baastrup’s disease. As differential diagnoses were ruled out and conservative measures were exhausted, the indication for surgical decompression was made. The particularly invasive treatment of Baastrup’s syndrome is an ongoing topic of discussion. Only a few case studies and reports address the surgical treatment options for Baastrup’s disease (see Table 1).

**Table 1:** Literature review of surgical treatment in Baastrup’s disease.

Category	Year	Authors	Patients (n)	Surgical technique	Outcomes
Resective	1944	Franck et al. [14]	10	Partial resection	Complete recovery: 4 patients. Improvement: 4 patients. Remaining complaints: 2 patients
	1989	Beks et al. [15]	64	Partial resection and full resection	Complete recovery: 11 patients. Remaining/returning complaints: 53 patients
	2015	Yue et al. [16]	8	Partial resection	Complete recovery: 8 patients
	2019	Kerroum et al. [17]	1	Full resection	Complete recovery: 1 patient
	2021	Lin et al. [18]	3	Endoscopic interspinous plasty (partial resection)	Improvement: 3 patients
Non-resective	2018	Mostofi et al. [19]	47	Interlaminar lumbar device implantation	Improvement: 45 patients. Delayed improvement: 1 patient. No improvement: 1 patient
	2018	Clark et al. [20]	1	Interspinous radiofrequency lesioning	Complete recovery: 1 patient

First, a distinction must be made between resective and non-resective surgical techniques, the latter involving the placement of interlaminar devices or radiofrequency ablations, which are not intended to be the subject of this discussion but should be considered surgical alternatives.

In 1944, Franck et al. initially described the partial resection of the spinous processes performed in a total of 10 patients [14]. Complete recovery was found in 40%, an clinical improvement in another 40%, and an unchanged condition in only 20%. Of interest, patients who did not respond to therapy presented with confounding comorbidities. As such, one patient presented with lumbalization and spina bifida. Another patient previously suffered from a wedge compression fracture of T12, whereas the partial resection of the spinous processes was performed at the directly adjacent levels of L1-L3 [14]. The highest number of case series was reported by Beks et al., who performed either partial or total resection of the spinous processes in 64 patients with Baastrup’s disease, from which only 11 (17%) patients benefited [15]. However, all patients revealed concomitant pathological spinal changes (e.g., disc degeneration, congenital malformations, spinal stenosis, and lumbar spondylosis), so similar to Franck et al., additional comorbidities were present, thereby possibly affecting the poorer postoperative outcome.

Other studies continue to demonstrate the value of either partial or complete resection of the spinous processes leading to a complete recovery with a follow-up of up to 2.5 years [16,17]. As a minimally invasive approach, an endoscopic partial resection demonstrated clinical improvements in the visual analog scale and Oswestry disability index scores [18]. None of the studies showed surgically associated complications. Likewise, we could not observe any complications after intervention in our patient.

In summary, besides a lack of interventional studies, a precise indication for surgical resection is essential for patient-centered outcomes. Comorbid factors or pathologies must either be excluded or prioritized to achieve optimal patient outcomes. In our patient, we had the advantage that an isolated Baastrup’s disease was present without any other concomitant diseases that could affect the clinical outcome.

## Conclusions

Baastrup’s disease is a frequently observed radiological phenomenon, mainly in the elderly, wherein the spinous processes of adjacent vertebrae form pseudarthrosis. A distinction must be made between the solely radiological sign and the actual clinically relevant pain syndrome, the latter of which should be considered in the differential diagnosis of back pain. Although no precise therapeutic guidelines exist, pain control by conservative measures is obligatory. Thus, analgesic therapy, physiotherapy, and interspinous injections establish the initial therapy of choice. However, if clinical evidence of Baastrup’s disease exists, differential diagnoses have been ruled out, and conservative measures have been exhausted, surgical decompression may offer a low-risk and beneficial therapeutic option, as shown in this patient’s case.
